# The Role of Epicardial Adipose Tissue in the Development of Atrial Fibrillation, Coronary Artery Disease and Chronic Heart Failure in the Context of Obesity and Type 2 Diabetes Mellitus: A Narrative Review

**DOI:** 10.3390/jcdd9070217

**Published:** 2022-07-05

**Authors:** Anirudh Krishnan, Harman Sharma, Daniel Yuan, Alexandra F. Trollope, Lisa Chilton

**Affiliations:** 1 College of Medicine and Dentistry, James Cook University, Townsville, QLD 4811, Australia; anirudh.krishnan@my.jcu.edu.au (A.K.); harman.sharma@my.jcu.edu.au (H.S.); daniel.yuan@my.jcu.edu.au (D.Y.); 2 Centre for Molecular Therapeutics, Australian Institute of Tropical Health and Medicine, College of Medicine and Dentistry, James Cook University, Townsville, QLD 4811, Australia; alexandra.trollope@jcu.edu.au; 3 Centre for Molecular Therapeutics, Australian Institute of Tropical Health and Medicine, College of Public Health, Medical and Veterinary Sciences, James Cook University, Townsville, QLD 4811, Australia

**Keywords:** adipokines, fibrofatty infiltrates, inflammatory adipose phenotype, intramyocardial fat

## Abstract

Cardiovascular diseases (CVDs) are a significant burden globally and are especially prevalent in obese and/or diabetic populations. Epicardial adipose tissue (EAT) surrounding the heart has been implicated in the development of CVDs as EAT can shift from a protective to a maladaptive phenotype in diseased states. In diabetic and obese patients, an elevated EAT mass both secretes pro-fibrotic/pro-inflammatory adipokines and forms intramyocardial fibrofatty infiltrates. This narrative review considers the proposed pathophysiological roles of EAT in CVDs. Diabetes is associated with a disordered energy utilization in the heart, which promotes intramyocardial fat and structural remodeling. Fibrofatty infiltrates are associated with abnormal cardiomyocyte calcium handling and repolarization, increasing the probability of afterdepolarizations. The inflammatory phenotype also promotes lateralization of connexin (Cx) proteins, undermining unidirectional conduction. These changes are associated with conduction heterogeneity, together creating a substrate for atrial fibrillation (AF). EAT is also strongly implicated in coronary artery disease (CAD); inflammatory adipokines from peri-vascular fat can modulate intra-luminal homeostasis through an “outside-to-inside” mechanism. EAT is also a significant source of sympathetic neurotransmitters, which promote progressive diastolic dysfunction with eventual cardiac failure. Further investigations on the behavior of EAT in diabetic/obese patients with CVD could help elucidate the pathogenesis and uncover potential therapeutic targets.

## 1. Introduction

Obesity is defined as a body mass index (BMI) above 30 and is characterized by excess storage of adipose tissue (AT) in the body, such as around the viscera, within the bone marrow, within the muscle, or subcutaneously [1]. Worldwide, obesity rates have been on a steady incline [2]. This is particularly significant in low- and middle-income countries, as high-fat, energy-dense, but micronutrient-poor foods are cheaper and easier to obtain [2]. Obesity is strongly correlated with type 2 diabetes mellitus (T2DM), with up to 85% of diabetics being overweight/obese [3]. CVDs are a group of disorders affecting the functioning of the heart. They remain the most significant comorbidity in the obese and diabetic populations and cause 17.9 million deaths annually worldwide [4].

T2DM is a condition characterized by insulin resistance. Insulin sensitivity is prone to fluctuation throughout life, affected by exercise, carbohydrate intake, and age, but a pathological level of resistance results in chronic hyperglycemia and is the cause of numerous complications [5]. The American Academy of Family Physicians (AAFP) describes fasting blood glucose of >7 mmol/L or an HbA1c of >6.5% as diagnostic of T2DM [6]. Insulin resistance has been associated with AT, which has been shown to influence metabolism through the release of leptin, adiponectin, cytokines, and other proinflammatory substances that can alter central appetite signaling and energy utilization at a cellular level [5]. Collectively, these bioactive molecules are called adipokines. Excess adiposity is thought to activate stress signaling, leading to chronically elevated pro-inflammatory kinase pathways that reduce cellular response to insulin [7]. Non-esterified fatty acids (NEFAs) are another endocrine factor released from AT that promote insulin resistance and tend to be elevated in obese or T2DM patients [5]. Although truncal obesity is a greater CVD risk factor, studies have long suggested that visceral fat has greater lipolytic properties and thus, can play a greater role in the development of insulin resistance than peripheral fat deposits [8].

EAT is a visceral fat depot, forming part of the AT surrounding the heart. It has recently gained attention for its suspected role in the development of CVD, in the setting of dysregulated pro-inflammatory adipokine secretions. T2DM and obesity are themselves associated with a high CVD risk due to their associations with dyslipidemia, hypertension, and hyperglycemia [9]. It is likely that a pathologic phenotype of EAT is associated with the development and propagation of these disease states.

The general aim of this review is to investigate the relationship between EAT and associated CVDs, specifically, AF, CAD, and chronic heart failure (CHF), and their pathophysiological mechanisms in the context of obesity and T2DM. EAT has rapidly gained attention in the past decade for its role in CVD, with new insights being postulated frequently. Reviewing the current understanding of the role of EAT in these diseases would be illuminating in determining future directions for newer studies in this field and for novel therapies to be developed.

## 2. Physiology of Epicardial Adipose Tissue

### 2.1. Origin, Distribution, and Anatomy of EAT

**Figure 1:**
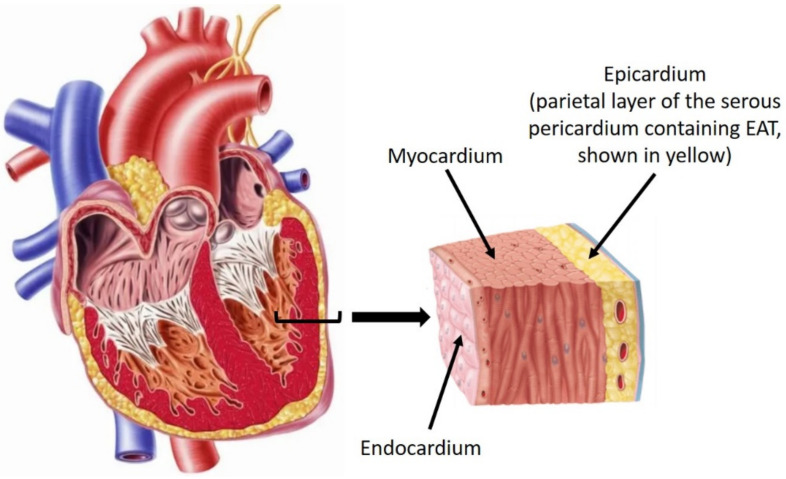
Layers of tissue surrounding the heart. EAT is situated deep in the pericardial fat, between the layers of visceral pericardium and myocardium. Pericardial fat is situated between the visceral and parietal pericardium. Paracardial fat is the general term referring to fat associated with the heart. Adapted from Newman (**left**) [13] and Betts et al. (**right**) [14].

EAT forms part of the visceral fat depot that surrounds the heart and the great vessels. It is located inferior to the visceral pericardium and is in direct contact with the myocardium (Figure 1). It is separate from pericardial fat located external to the parietal pericardium, which is also a component of the paracardial fat depot [10,11]. These definitions are of importance as they are sometimes used interchangeably within the literature. In humans, EAT derives from the splanchnopleuric mesoderm, along with omental and mesenteric fat. EAT is composed of adipocytes, ganglionated nodes, and stromal and immune cells. It has a typical spread across the right ventricular (RV) free wall, the anterior surface of the left ventricle, and the interventricular and atrioventricular grooves [11,12]. It receives its vascular supply from the coronary arteries and also forms the part of the perivascular adventitia. It is thought to play a protective mechanical role against the tension and twist of an arterial pulse [11,12]. Similarly, its cover over the ventricular wall allows an overall cushioning effect against mechanical forces on the thorax.

**Figure 2:**
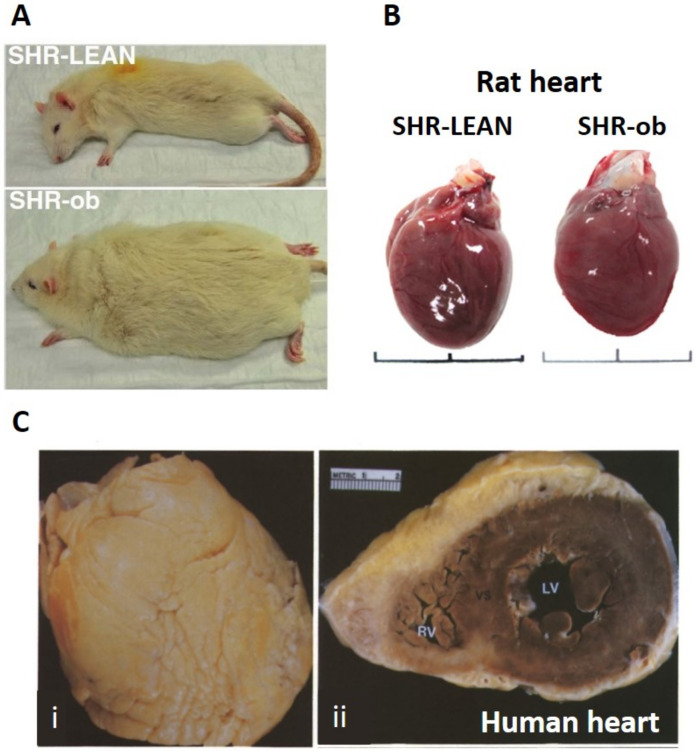
EAT thickness between lean and obese rats in panels (**A**,**B**) compared to EAT thickness found on the heart of an 83-year-old overweight female in panel (**C**). Reproduced from Krishnan et al. [17] under the Creative Commons Attribution License.

EAT, on average and in physiologic conditions, accounts for 20% of cardiac weight in humans [15]. There is, however, a variance in EAT characteristics between species. Rats and other rodents tend to have minimal EAT, while larger mammals have EAT surrounding the atria, coronary vasculature, and ventricular surface (Figure 2) [16].

### 2.2. Brown vs. White Adipose Tissue and Cardiometabolic Modulation

AT can be classified as white or brown depending on its function. Human fat is predominantly white AT, but small quantities of brown AT can also be found (particularly in infants) [11,18]. Brown AT aids in thermogenesis due to high concentrations of mitochondria specialized in heat production through the expression of thermogenic genes such as unique uncoupling protein (UCP)1 or peroxisome proliferator-activated receptor-gamma coactivator-1alpha (PGC1α) on the inner mitochondrial membrane. The protein facilitates the release of chemical energy as heat through the consumption of glucose and lipids [18,19]. EAT displays brown-like characteristics in healthy humans, protecting the heart in times of hypothermia or ischemia through its regulation of cardiac metabolism and secretion of cardioprotective adipokines [11]. This is in contrast to white fat, which mainly acts as an energy reserve and endocrine system influencing insulin sensitivity without thermogenesis [11,18].

Additionally, EAT regulates the cardiometabolic profile through the release of free fatty acids (FFA) in times of demand. EAT, unlike subcutaneous AT, has higher rates of insulin-induced lipogenesis and lipolysis as FFAs are the primary source of energy for cardiomyocytes [11,20]. This also allows it to act as a buffer system against toxic levels of FFAs which can cause a pro-inflammatory reaction in the tissue. EAT differs from subcutaneous AT in that the latter has higher levels of triacylglycerols while the former has higher levels of glycerophosphatidylcholines, suggesting more stable lipid droplets in EAT [21]. EAT is also in direct contact with the myocardium, which allows it to exert both paracrine and endocrine influences on the cardiomyocytes and vascular endothelium of coronary arteries through the adipokines and other chemical messengers [22].

### 2.3. EAT Secretome and Adipokines

EAT is an active secretory tissue that regulates cardiometabolism, and inflammatory and vascular responses [23]. A balance of pro-inflammatory and anti-inflammatory adipokines is normally maintained, generally favoring an anti-inflammatory state [11]. When there is low oxidative stress, EAT expresses adiponectin, an adipokine that increases insulin sensitivity and is anti-atherogenic/anti-inflammatory. It also protects vascular endothelium by sustaining endothelial nitric oxide synthase-dependent pathways and limits fibrosis in the myocardium [24]. Other adipokines such as omentin and apelin provide similar antioxidant and anti-inflammatory effects. In contrast, adipokines such as tumor necrosis factor-alpha (TNF-α), visfatin, and resistin can increase lipolysis, causing an increased release of NEFAs [25]. Mediators released from inflammatory cells such as interleukin (IL)-6, transforming growth factor-beta (TGF-β) and angiotensin II (AngII) can increase leukocyte recruitment, fibroblast proliferation, and collagen production [11,24]. While these are necessary processes in the context of an acute inflammatory state, evidence has emerged that they can be inappropriately exaggerated in conditions such as T2DM. This is discussed further in Section 4.

### 2.4. Diagnostic Imaging Characterizing the Thickness and Volume of EAT

In the context of disease, the structural phenotype of the EAT is an equally important factor to be considered. Quantifying EAT thickness and volume is typically performed through imaging techniques, such as computed tomography (CT), echocardiography, or cardiac magnetic resonance imaging (MRI). The maximal thickness is typically noted on the RV-free wall on echocardiography [26]. No consensus has been reached regarding a “normal” EAT thickness, but trends associated with the disease have been recognized, particularly with increasing EAT thicknesses [26].

Transthoracic echocardiography is especially accurate in determining EAT thickness, but a 2D study would not be accurate in determining EAT volume [27]. However, it does hold benefits in being a bedside investigation that is cost-effective and is without the risk of radiation exposure. However, the gold standard remains MRI due to the image resolution, volumetric analysis, and fat + water separation that allows for distinction among the soft tissues. Similarly, CT scans provide the option of volumetric assessment and are less expensive than MRIs [26]. However, it is important to note that quantification of thickness and volume of EAT remains a challenge due to external factors such as interference from heartbeats, fat droplets, and variations in water content [22]. No large-scale trials have been conducted to detect sensitivities and specificities of imaging modalities to detecting EAT thickness and volume.

## 3. Pathophysiologic States of Epicardial Adipose Tissue

Several studies exist comparing EAT thickness and epicardial fat volume (EFV) in diabetic and non-diabetic patients through non-invasive methods. EAT thickness, when assessed with echocardiography, was on average 5.4 mm (IQR-4.2–7.4 mm) in diabetic patients (*n* = 40) compared to 3.9 mm (IQR-2.9–4.8 mm) in the control group (*n* = 281) [28]. Using CT, EFV in diabetic patients (*n* = 215) was averaged to be 112.9 mL (IQR-21.4–442.2 mL) compared to 82.6 mL (IQR-11.3–318 mL) in the control group (*n* = 381) [22,29]. This demonstrates that T2DM patients have on average significantly thicker and more voluminous EAT. It is worth noting that these studies did not correct for BMI/waist circumference (WC), meaning obesity could have also been a factor causing the elevated EAT levels. Similarly, patients with metabolic syndrome (MS) had, on average, a two-fold greater EAT thickness compared to those without MS, with the most significant association being the indices of obesity [30]. Patients with EAT thickness >5 mm and MS also had a 12-times greater risk of developing T2DM compared to those with EAT thickness <5 mm and normal glucose tolerance [28]. While MS itself is associated with a 3.5–5.2 times greater relative risk of developing T2DM than in the control population [31], one must account for the additional risk associated with the elevated EAT thickness. Verma et al. also noted that systolic EAT thickness of >5 mm and diastolic EAT thickness of >4 mm on echocardiography had sensitivities of 85% and 83%, and specificities of 70% and 72%, respectively, in predicting CAD [32]. EAT is likely intimately associated with the development of T2DM and could play a role in perpetuating insulin resistance in these patients. Correspondingly, weight loss in patients through exercise or bariatric surgery has been shown to reduce EAT thickness [15]. Peripheral adiposity and EAT thickness thus appear to be linked with both contributing to the development of T2DM. This finding suggests that weight loss could reduce the volume of EAT itself, but the question remains if this would be equally beneficial towards developing glucose tolerance independently.

EAT has been demonstrated to upregulate inflammation in disease states such as T2DM, obesity, and CAD. Increased expression of pro-inflammatory cytokines such as IL-6 and TNF-α have been demonstrated in situ, as well as chronic inflammatory cells in the perivascular EAT (PVAT) of patients with coronary atherosclerosis [33]. IL-6, visfatin [33], and leptin levels are also shown to be increased with larger EAT volumes, with lower anti-inflammatory cytokine levels, such as adiponectin and adrenomedullin [11,20]. The roles of these cytokines have been expanded upon in Table 1. This indicates that the EAT secretome can become inflammatory [34] and atherogenic in its profile, with a diminishing of protective factors; this secretome may disrupt cardiac metabolism. Adiponectin is involved with the synthesis of nitric oxide as mentioned earlier. Decreased levels of adiponectin lead to coronary vasoconstriction and defective endothelial function [35,36]. Adipokines such as TNF-α have also been shown to precipitate cardiomyocyte apoptosis, resulting in replacement fibrosis. Replacement fibrosis tends to be more disruptive to electrical conduction than reactive fibrosis as they alter the electroanatomical structure by replacing dead cardiomyocytes with extracellular matrix (ECM) [37], compared to the latter where there is an expansion of ECM without disturbing cardiomyocyte communication. Pro-inflammatory cytokines can also directly promote fibroblast maturation and differentiation into myofibroblasts [38]. This is significant as myofibroblasts deposit collagen at a much higher rate than fibroblasts, leading to fibrosis [39].

Together, the increased collagen content in the heart can lead to arrhythmias and affect contractility. Ng et al. demonstrated interstitial fibrosis and increased myocardial fat accumulation associated with increased EAT volume and T2DM, utilizing an MRI sequence with fat and water separation [44]. Intramyocardial fat was a physiological phenomenon, commonly noted in the elderly and those with elevated visceral adiposity. [12,45]. Increased intramyocardial fat accumulation was also noted, using echocardiography and autopsy studies in patients with increased EAT and was particularly evident in the RV [46]. Multiple causes have been hypothesized for its etiology, a prominent one being that accumulation of EAT results in the migration of adipocytes to the myocardium [12]. This is often related to replacement fibrosis due to active inflammatory processes and a disturbed cardiometabolic profile (such as in T2DM or obesity). In diabetic patients who have been shown to have increased EFVs with an increased inflammatory profile, the myocardial adipose infiltrates also become inflamed and fibrotic. Such infiltrates are termed “fibrofatty”. These fibrofatty infiltrates can be pathological when excessive, causing cardiac mechanical disturbances and playing a role in non-ischemic cardiomyopathies [12,47]. Myocardial fibrofatty infiltrates have also been linked to increased lipolysis and the maintenance of a high FFA state in the EAT, which is suggested to occur as a phenotypic shift from brown-like to white AT [18,47]. This is significant as it indicates EAT characteristics shift from being cardioprotective to maladaptive in T2DM and obesity. Changes specific to T2DM have also been noted, with greater pro-inflammatory macrophage infiltration and greater expression of scavenger receptors, such as lectin-like oxidized low-density lipoprotein (LDL) receptor (LOX)-1, in the EAT when compared to non-diabetics [48]. Other studies have shown a cluster of differentiation (CD)8 cytotoxic T cell infiltration in the AT of obese patients [49]. This is in keeping with the hypothesis that EAT undergoes pathologic changes in T2DM and obesity. Now, the question remains: how do these changes correlate to an increased risk of CVDs?

### 3.1. Epicardial Adipose Tissue and Atrial Fibrillation

Intercellular communication between cardiomyocytes occurs through gap junctions which consist of Cx proteins, mainly Cx40, Cx43, and Cx45. Cx40 is the most common atrial connexin. Cx43 is expressed predominantly in the intercalated discs of ventricular myocytes, while Cx45 is limited to the nodal and conductive tissue [50,51]. The intercalated discs, normally located at the ends of cardiomyocytes, allow for unidirectional electrical conduction across the myocardium. AF is the most common sustained arrhythmia affecting the general population and significantly contributes to cardiovascular morbidity and mortality [52]. It tends to have a natural progression of severity from intermittent to paroxysmal which is defined as lasting <7 days, before becoming persistent and lasting >7 days or requiring cardioversion [53]. Long-standing AF is defined as sustained fibrillation lasting >1 year and AF is considered permanent when the patient and physician reach a decision to cease rhythm-correcting therapy [53]. Obesity is a recognized risk factor for AF, with meta-analyses demonstrating a 49% greater risk of AF development in these populations [52]. Other studies have also demonstrated that >10% of body weight loss through diet and exercise reversed the progression of AF from persistent to paroxysmal/absent in 88% of patients [54]. The causes of AF are varied as some occur spontaneously, while others are related to structural heart diseases such as atrial enlargement or myopathy. In the cases of spontaneous AF, it is theorized that a pathologic substrate leads to the development of focal, ectopic stimuli from a point in the atria that overrides the sinus rhythm [55]. These ectopic foci can manifest initially as early afterdepolarizations (EADs) or delayed afterdepolarizations (DADs) [38,56]. Such repeated triggered activity often arises near the pulmonary veins, leading to a persistent arrhythmia. In cases of structural heart disease, the electroanatomical remodeling results in altered action potential (AP) conduction velocity and refractory periods, allowing for re-entry loops to occur, which may lead to AF [34,56,57].

Ectopic AP production from cardiomyocytes is associated with changes in intracellular calcium handling. Cardiac calcium homeostasis is beyond the scope of this review; the reader is directed to recent thorough reviews [58,59]. EADs may arise when AP duration is prolonged, allowing inward depolarizing currents due to recovery from inactivation of voltage-gated L-type calcium channels and/or the production of a sodium current via the sodium–calcium exchanger. DADs may arise when ryanodine receptors spontaneously release calcium from the sarcoplasmic reticulum when cardiomyocytes are at resting membrane potential [58,59]. Variability in AP duration in cardiomyocytes within the myocardium may give rise to wavebreaks, where some waves of depolarization experience conduction block due to refractory cardiomyocytes while others propagate, allowing re-entry to develop [58]. The probability of such re-entry developing is also increased with structural modification of the atria, such as fibrosis separating strands of cardiomyocytes [17,58,59].

**Figure 3:**
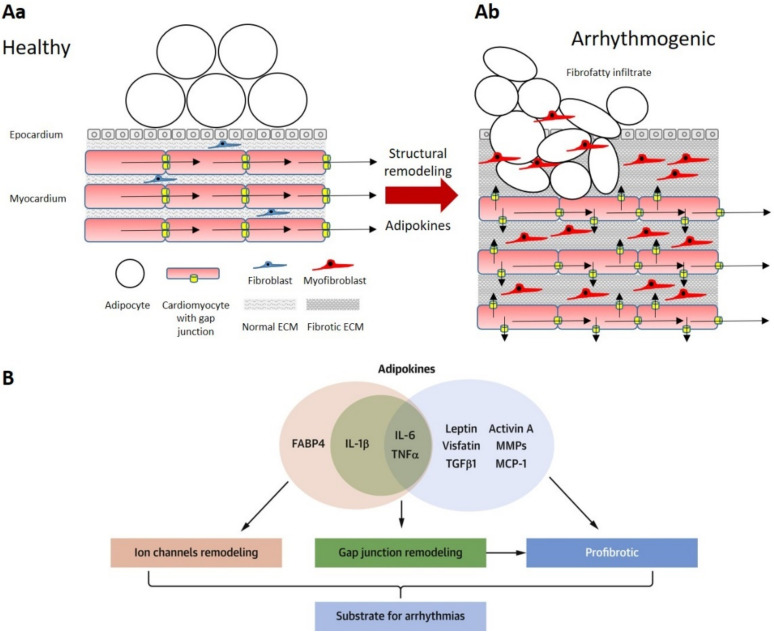
Formation of arrhythmogenic substrate as a result of fibrofatty infiltrates increasing conduction heterogeneity across the atrial myocardium. Panel (**Aa**): Action potential (AP) conduction is linear in healthy myocardium, where the endomysium is comprised of a thin layer of connective tissue with fibroblasts and EAT is predominantly supramyocardial. In contrast, with fibrofatty infiltration of the myocardium, fibrosis, structural remodeling, and production of pro-inflammatory and pro-fibrotic adipokines (Panel (**Ab**)), AP conduction becomes ‘zigzag’ and the myocardium is at risk of arrhythmias. Examples of specific adipokines associated with arrhythmogenic remodeling are shown in Panel (**B**); FABP4 = FA binding protein 4, IL = interleukin, TNF = tumor necrosis factor, TGF = transforming growth factor, MMP = matrix metalloproteinases, MCP = monocyte chemoattractant protein. Panel (**B**) reproduced from Ernault et al. [34] under the Creative Commons CC-BY-NC-ND license.

Local infiltration of the myocardium by EAT can hinder the cardiac conductive system by separating strands of cardiomyocytes, as well as disrupting intercellular communication between cardiomyocytes and causing an anatomical block of AP conduction. These structural changes, in conjunction with the increased risk of ectopic foci in cardiomyocytes surrounding fibrofatty infiltrates, may give rise to re-entry loops, sustaining AF [48,60]. Ernault et al. noted from autopsy studies that myocardial fat infiltrates are typically organized as thin cords extending from the subepicardial layer and in-between cardiac bundles or amidst large areas of fibrosis [34]. The Framingham Heart Offspring Study found that pericardial fat predicted AF risk with an odds ratio of 1.28 for every standard deviation (SD) rise in fat volume when assessed through CT [61]. This study also noted that P wave (atrial depolarization) duration was positively correlated with EAT thickness [34]. Other studies have similarly found that with each 10 mL increase in EFV, there was a 13% greater risk of AF following adjustment for other risk factors and that patients with persistent AF consistently had thicker EAT than those with paroxysmal or no AF [62,63]. High EFVs were also associated with a strong risk of recurrence following AF ablation therapy, suggesting a significant role in the development of AF, and/or its perpetuation [64]. Haemers et al. found that AF was associated with fibrosis of subepicardial fat in human hearts and in sheep hearts with rapid atrial pacing, similar to the previously described fibrofatty infiltrates seen in obese and diabetic patients [65]. Fewer mouse/rodent models exist investigating EAT as it tends to be minimal on the hearts of small mammals, compared to large mammals (refer to Figure 2) [16]. Experimental models with dogs demonstrated fibrotic remodeling of the atrial myocardium associated with sustained atrial tachycardia in the context of CHF [66]. These data suggest that fibrosis may independently create substrates for AF in both the EAT and fibrofatty infiltrates within the myocardium and that atrial tachyarrhythmias could worsen (Figure 3) [56]. Increased inflammation was also recognized in the EAT, with CD8 T cell infiltrates and lymphoid aggregates in the fibrotic infiltrates, mainly bordering on the areas between adipocytes and fibrosis [65]. Sheep exposed to high-calorie diets and progressive weight gain displayed similar atrial electroanatomical remodeling changes to those suggested by human clinical studies; specifically, increased atrial volumes, atrial interstitial fibrosis/lipidosis, inflammation, and increase in interstitial and cytoplasmic TGF-β levels. The histologic analysis also demonstrated greater perivascular collagen deposition in obese sheep compared to baseline [67]. Therefore, it is clear that fibrofatty infiltrates are a common characteristic in AF patients and that these infiltrates tend to be associated with inflammation of the EAT and atrial remodeling leading to an arrhythmogenic substrate.

**Figure 4:**
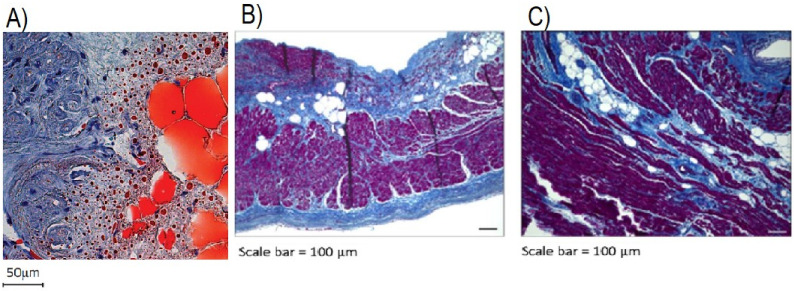
(**A**): Oil-red-O-stained RAA sections depicting heterogenous interface between infiltrating adipose tissue (red, globular cells) and myocardium (blue). (**B**,**C**): LAA sections stained with HE shows significant EAT infiltration (empty, globular cells) with fibrosis (blue strands) noted along adipocytes. Myocardium is represented as magenta. (**A**) Reproduced with permission from Nalliah et al. [68]; (**B**,**C**) reproduced with permission from Abe et al. [69].

Studies have noted patients with AF displayed significant fatty infiltrates originating from the subepicardium in both left and right atrial appendages (LAA and RAA), with some evidence of transmural infiltration (Figure 4). AF was also associated with increased fibrotic content in the myocardium, which showed a strong positive correlation between atrial EAT volume and fibrosis [68,69]. This is similar to previous findings where progressive fibrosis of AT infiltrates was noted in permanent AF patients [61]. Venteclef et al. reported upregulated activity of matrix metalloproteinases (MMPs) and adipokine activin A, a subset in the family of TGF-β cytokines, in pathologic conditions leading to myocardial fibrosis [70]. Similarly, activin A expression in EAT was found to be an independent risk factor for postoperative AF [71].

**Figure 5:**
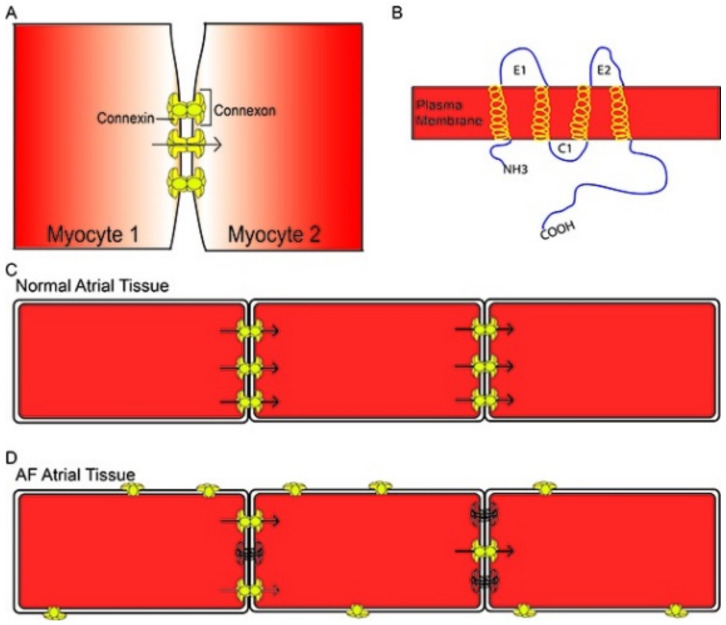
(**A**): Graphical depiction of myocyte-to-myocyte communication through connexin proteins at intercalated discs located at the ends of the cells. (**B**): Graphical depiction of structure of connexin protein in relation to plasma membrane. (**C**): Connexin distribution in normal atrial tissue. (**D**): Lateralization of Cx proteins away from intercalated discs, leading to aberrant communication between cardiomyocytes. Reproduced from Jennings and Donahue [56] under a Creative Commons Attribution 3.0 Unported License.

Another proposed factor in the occurrence of arrhythmias is the lateralization of sarcolemmal Cx43 proteins away from the intercalated discs [72,73]. Stresses such as ischemia or inflammation promote phosphorylation of Cxs leading to their lateralization across the cell membrane [56]. This redistribution affects the directional flow of current and causes heterogeneity of electrical conduction across the atria. In combination, these changes would provide a substrate for re-entry loops and AF [56]. Nalliah et al. reported that increased local EAT content in the right atrium was associated with increased cardiac fibrosis, complex activation patterns, and lateralization of cardiomyocyte Cx40 gap junction proteins (as demonstrated in Figure 3(Ab) and Figure 5) [68].

Complex fractionated atrial electrograms (CFAEs) are thought to indicate areas of conduction slowing or blocking and high-frequency electrical sources in the myocardium, such as ectopic pacemaker activity [74]. Areas of CFAEs correlated significantly with local pericardial fat volume in patients with AF [75]. Some CFAEs were also localized in relation to the ganglionated plexi present in EAT. Other studies have also reported the paracrine mediators secreted from EAT had similar effects of conduction slowing and AP prolongation [76], suggesting more pathogenic mechanisms than purely mechanical. However, other reports suggest that AP duration is reduced, which is considered a more common pathological change in the development of AF [58,59,77]. For example, Martinez-Mateu et al. reported that in the atria of obese guinea pigs fed a high-fat diet, rapid pacing was associated with reduced voltage-gated potassium and L-type calcium current, with shortened AP duration and increased incident of afterdepolarizations [78].

A novel biomarker, YKL-40, has emerged recently as an indicator of fibrosis and cardiovascular disease. It is an inflammatory glycoprotein expressed by several cell types, such as vascular smooth muscle cells and macrophages, which plays a key role in fibroblast proliferation and ECM deposition [79]. Studies have shown plasma YKL-40 levels to be elevated in patients with T2DM, independent of parameters of obesity [80]. YKL-40 mRNA levels were elevated in EAT, when compared to subcutaneous AT, and elevated in AF patients when compared to patients with normal sinus rhythm. YKL-40 expression levels also showed a linear, and potentially causative, relationship with the extent of collagen I volume in the atrial myocardium in AF patients [79].

Inflammasomes have also been found to play a role in AF such as the leucine-rich repeat-containing receptor (NLR) family pyrin domain-containing 3 (NLRP3) inflammasome. A study conducted by Yao et al. [81], demonstrated increased activity of NLRP3 inflammasomes in human atrial cardiomyocytes and that it was associated with pathogenesis AF. The NLRP3 inflammasome is associated with cardiomyocyte sarcoplasmic reticulum calcium release during diastole in mice, which may predispose the cardiomyocytes to produce DADs. AP duration was also reduced, which may promote and maintain re-entry in AF [82]. These studies have shown the importance of the NLRP3 inflammasome in the pathogenesis of AF and identifies the inflammasome as a potential therapeutic target.

In addition to the previously mentioned pathogenic mechanisms, tissue culture studies by Lin et al. demonstrated that when epicardial adipocytes were incubated with atrial cardiomyocytes, cardiomyocytes had longer AP durations, higher resting membrane potentials, larger late sodium currents, transient outward potassium currents, and L-type calcium currents, but smaller inward rectifier potassium currents than control cardiomyocytes. The most significant of these changes was the increase in late sodium currents. Increased late sodium currents have the most arrhythmogenic potential, prolonging the AP duration and allowing for afterdepolarizations to occur [83]. Increased incidence of triggered ectopic activity is associated with both paroxysmal and sustained AF [58]. FFAs are also thought to have effects on conduction [84]. Interestingly, the supernatant of these adipocytes was isolated and added to the cardiomyocytes and showed similar AP duration prolongation when used on LA myocytes [85]. This suggests that paracrine mediators and adipokines released by EAT influence cardiomyocyte ion channels, and intracellular calcium handling, and affect AP durations. Intracellular signaling controlling cardiomyocyte calcium handling affected by and underlying cell electrophysiology is complex and changes in disease states. Cardiomyocyte calcium handling has been extensively studied [58,59]. The cellular mechanisms by which EAT modulates cardiomyocyte calcium handling, indirectly by adipokines or directly by structural changes, are not as well studied. The reader is referred to Antonopoulos and Antoniades [11] for a detailed review of the current understanding of cellular mechanisms within EAT, cardiomyocytes, and fibroblasts within the myocardium and Gawałko et al. [77] for a detailed review of the mechanistic changes underlying AF in obese patients.

Further evidence to the local effect of EAT on cardiac electrophysiology was that conduction abnormalities in obese patients were most significant in the posterior LA, where EAT thickness was greatest and where the fat depots are almost contiguous with the myocardium, and hence, in especially close proximity to it [86]. This suggests the physical infiltration of EAT into the myocardium as well as paracrine effects on AP conduction. This is a useful finding as a potential target for therapy in preventing EAD/DAD or re-entry loop occurrence.

### 3.2. Epicardial Adipose Tissue and Coronary Artery Disease

CAD is the most common form of cardiovascular disease in Australia, currently affecting over 580,000 individuals [87]. An important Finnish study first indicated that diabetic patients increased the 7-year risk of myocardial infarction secondary to CAD [88]. T2DM is characterized by microvascular and macrovascular complications, the latter of which in this case is atherosclerosis. It is a progressive condition characterized by occlusion of the coronary arteries by atherosclerotic plaques. It is especially common in patients with T2DM, affecting up to 30% [89]. This condition can eventually lead to myocardial ischemia and can result in infarctions, ischemic cardiomyopathy, and heart failure [90]. Atherosclerotic plaque formation occurs due to a cascade of events, the precipitant being damage to the vascular intimal lining. This leads to the accumulation of circulating LDLs which causes a low-grade inflammation as macrophages infiltrate the media to phagocytose the invading material, forming foam cells. The macrophages release pro-fibrotic and other pro-inflammatory factors which also trigger oxidative stress through reactive oxygen species (ROS). The cycle of inflammation and infiltration leads to the formation of fatty streaks, and eventually, plaques [91]. In diabetic patients, this process is accelerated due to the glycation and oxidation of LDLs, which make them increasingly atherogenic, which is combined with hypertriglyceridemia due to reduced insulin action and dyslipidemia [92,93].

The NLRP3 inflammasome has also been found to play a role in atherosclerosis [94]. Elevated levels of components of the NLRP3 inflammasome have been found in atherosclerotic plaques and have been shown to play a role in the initiation and progression of atherosclerosis [95]. In addition to this, NLRP3 expression has also been positively correlated with epicardial adipose tissue volume, whilst adiponectin expression was negatively correlated, and it has been suggested that local stimulation of NLRP3 inflammasome can stimulate EAT [94].

Obesity is one of the most significantly referenced cardiac risk factors, but newer studies suggest a complex relationship between obesity and CAD [11,96]. Subcutaneous fat in the thighs and hips is primarily white fat and has long since been known to have little effect on CAD risk, while visceral adipose tissue (VAT) has been strongly associated with an increased cardiometabolic risk [97]. The “obesity paradox” is the finding that while obese BMIs are associated with increased CVD risk, lean BMIs correlate with an increased risk of future CVD events in patients with existing CAD or CHF [11,96]. EAT is a type of VAT and shares similarities in its metabolic profile with other visceral fat depots. Hence, it is intrinsically separate from subcutaneous AT. The differences in distribution around the body could be a contributing factor to increased CVD risk if an individual tends to accumulate fat more viscerally than subcutaneously. Furthermore, there is ongoing evidence to suggest that CAD is associated with a brown to white transition of EAT, leading to a decrease in thermogenesis, increased ROS production, and an increasingly inflammatory secretome [18]. In addition, the under-expression of brown fat-like genes such as UCP1 and PGC1α has been demonstrated in the EAT of T2DM and CAD patients. Due to the role of these proteins in fatty acid oxidation and mitochondrial respiration, elevated levels of circulating triglycerides and lower high-density lipoprotein (HDL) levels suggest that the transition to white fat is an important occurrence in atherosclerotic disease [98,99]. However, it is still uncertain if this phenotypic change is the cause of T2DM/CAD disease states, or if it merely propagates the severity of the disease. Nonetheless, due to the ability of brown AT to oxidize large quantities of energy for thermogenesis, “re-browning” of AT has been postulated as a potential treatment goal to reduce cardiometabolic risks [19,100].

Further studies have expanded on the link between EAT and CAD. A prospective cohort study found EAT associated with elevated coronary artery calcium (CAC) scores, which are a marker used to predict future atherosclerotic risk in the vessel, as well as elevated plasma IL-8 levels [101]. A gender-specific role was also found, tending greater towards males, in the predictive capability of EAT for CVD [102]. Yerramasu et al. found a similar association with the severity and progression of CAC scores in T2DM patients without CVD [103]. There is some uncertainty regarding the exact significance of EAT in the formation of CAD as some studies have indicated that there is no correlation between coronary blood flow and myocardial perfusion [104,105]. Gullaksen et al. found no correlation between EFV (assessed by CT) and coronary plaque volume, although the authors acknowledge a small sample size and the cross-sectional nature of the study preventing a temporal relationship between EAT and CAD to be determined [106]. Even so, it seems a greater number of studies have demonstrated an increased risk profile for CVD associated with increased EAT. There is also uncertainty regarding the predictive capability of EAT for CVD risk over measures of peripheral adiposity such as WC/BMI, but some studies have demonstrated that measuring EFV/EAT thickness improves the accuracy of CVD incident risk and mortality models [102]. These findings together suggest that the pathophysiologic phenotype of EAT is perhaps more significant to the development of CAD than the absolute volume of EAT. This hypothesis is supported by the Cardiovascular RISk Prediction–Computed Tomography (CRISP–CT) study that indicated assessing inflammatory changes in PVAT significantly improved cardiac risk stratification when added to CAC and coronary CTA studies [107].

Mazurek et al. demonstrated infiltrating macrophages, mast cells, and CD3+ T cells in the PVAT surrounding coronary plaques [23]. The cellular infiltrate was also combined with elevated chemokine and cytokine levels such as IL-1B and IL-6. This study suggests an “outside-to-inside” mechanism whereby bioactive molecules arising from perivascular tissues influence vascular homeostasis within the lumen [23]. There is significant evidence to suggest an inflammatory phenotype involved in EAT and PVAT dysfunction, but the cause behind the altered phenotype has not been clearly determined. Obesity is associated with a rapid expansion of AT, which can often lead to hypoxic–ischaemic changes [108]. Elevated hypoxia-inducible factor (HIF)-1α protein levels suggest an induced angiogenic and fibrotic response, but one that cannot equally compensate for the tissue expansion [98]. HIF-1α has been linked to other pro-fibrotic cytokines, such as angiopoietin-related protein-2 (Angptl2), which have been shown to be elevated in EAT inflammation and fibrosis [65]. Additionally, the inflammatory reaction occurring from hypoxia induces further production of ECM. The microenvironment becomes pathologic with increased MMPs, myofibroblast differentiation, macrophage infiltration, reduced adiponectin, and collagen production that causes fibrosis of the EAT and PVAT which can then infiltrate into the myocardium [18,33]. The paracrine and endocrine effects of the EAT have also been positively correlated to clinical parameters such as carotid intima-media thickness as a result of atherosclerotic plaque [108]. There is also evidence that VAT fibrosis contributes to systemic metabolic effects such as glucose intolerance and dyslipidemia [108,109]. As a result of the inflammatory change in EAT and PVAT, dyslipidemia, and hypoxia, it appears coronary vasculature becomes more prone to endothelial dysfunction and lipid accumulation. Furthermore, Konwerski et al. [109] depicted a reduced EAT and PVAT volume (particularly around the left circumflex and left anterior descending arteries) on radio imaging in athletes compared to a sedentary population. Given the former population group also had a more favorable lipid profile, a hypothesis that the athletes had a reduced CVD risk could be drawn.

### 3.3. Epicardial Adipose Tissue and Chronic Heart Failure

CHF is defined as a failure of cardiac output to meet the metabolic requirements of the body. The clinical syndrome is characterized by dyspnea on exertion/rest, fatigue, signs of volume overload, and, in some cases, angina [110]. Its causes can be divided as structural or functional and can impair the ability of the ventricles to fill with blood or impair the contraction of the muscle to eject blood. CHF with reduced ejection fraction (HFrEF) is diagnosed when there is evidence of systolic dysfunction with ejection fraction (EF) < 45%, while CHF with preserved ejection fraction (HFpEF) is diagnosed when there is evidence of diastolic dysfunction with EF > 50% [111,112]. Patients with heart failure symptoms with EFs between these two ranges (40–49%) are categorized under CHF with midrange ejection fraction (HFmrEF), while CHF with recovered ejection fraction (HFrecEF) is a separate entity in patients who demonstrate a >10% improvement in EF after initial documentation of EF < 40% [111,112]. As detailed previously, T2DM patients are at an increased risk of CVD and can develop HFrEF through several pathogenic mechanisms. An acute thrombotic event in the context of CAD could result in an extensive myocardial infarct (MI) and affected ventricular function. Chronic CAD, on the other hand, could cause ischemic insult and micro-infarcts in the myocardium, resulting in an ischemic cardiomyopathy and consequently, HFrEF [91,113]. These entities are separate from diabetic cardiomyopathy, which is a CVD not attributable to known CAD, and one that has recently been of increasing concern as a consequence of T2DM in spite of appropriate CVD risk control [114]. Epidemiologic studies, such as Poirier et al., have shown evidence of subclinical LV diastolic dysfunction in 60% of T2DM patients [115]. However, it should be noted that they utilized Valsalva maneuvers during the imaging procedure which limits the ability to generalize these results as it might have screened a greater number of patients than in other studies. Nichols et al. performed larger retrospective studies that demonstrated that 14.1% of T2DM patients had underlying HFrEF/HFpEF and were 2.5 times more likely than non-diabetics to be diagnosed with CHF [116,117]. It is necessary to understand the prevalent risk of CHF in T2DM patients when assessing the impact EAT can have on the development of the disease.

Diabetic cardiomyopathy presents as a spectrum from subclinical diastolic dysfunction with LV hypertrophy initially, to symptomatic HFrEF eventually. Microvascular dysfunction has been attributed mainly to diabetic cardiomyopathy, which occurs as a result of advanced glycation end (AGE) products accumulating within the basement membrane of blood vessels (hyaline arteriolosclerosis) [91]. The UK Prospective Diabetes Study [118] found that a 12% rise in CHF risk was associated with a 1% rise in HbA1c and van Melle et al. noted a 36% increased risk [119]. The latter study had significantly fewer participants involved and only selected T2DM patients with stable CAD, while the former did not include that criterion. As a result, the difference in percentages could be attributable to a higher rate of HFpEF in pre-existing CVD patients. Nonetheless, hyperglycemia clearly has a role in increasing the risk for cardiomyopathy and appears to be proportionally associated with the severity and progression of HF. Impaired cardiac insulin signaling likely leads to a reduction in metabolic efficiency because of increased fatty acid utilization and internalization of the glucose transporter proteins (GLUT) [114]. Internalization of GLUT4 transporters leads to preferential localization of other transporters, such as CD36, on the sarcolemmal surface leading to increased fatty acid uptake. This combined with carnitine deficiency, which is a common metabolic disturbance in diabetics, leads to increased ROS production and inflammation as it is necessary for myocardial FFA utilization [120,121,122]. These factors together induce myocardial injury and stimulate AngII and other inflammatory cytokines to result in myocardial fibrosis. This correlates with the early manifestation of diastolic dysfunction due to cardiomyocyte stiffness [114].

Cardiac sympathetic hyperactivity is a hallmark of HFrEF/HFpEF that occurs as a compensatory mechanism to maintain cardiac contractility. In HF, under-perfusion of the kidneys leads to an upregulation of the renin–angiotensin–aldosterone system (RAAS) as physiologic compensation. Pathologically high mineralocorticoid levels have been associated with worsening cardiac insulin resistance and can worsen the morbidity and mortality of T2DM patients with CHF [114]. Angiotensin II is also known to be a potent fibrotic mediator and upregulation of AngII receptors is associated with cardiac remodeling leading to the diastolic dysfunction noted in diabetic cardiomyopathy [123]. Sympathetic nervous system (SNS) hyperactivation is also closely linked to the RAAS pathway and a hallmark of compensated HF. EAT contains both adrenergic and cholinergic neurons within ganglionic plexi that communicate with the extrinsic autonomic nervous system (ANS). Defective norepinephrine (NE) reuptake leads to a downregulation in cardiac NE transporters and reduced expression of neurotrophic factors [124,125]. Eventually, functional denervation of the heart occurs due to increased stimulation. Recent studies have shown EAT adds to this effect as an important and plentiful source of NE and could be propagating SNS hyperactivation and facilitating arrhythmias. Furthermore, it is hypothesized that EAT has a negative feedback effect on the cardiac sympathetic nerves, which leads to functional denervation of the heart [114,124]. This worsens the prognosis in CHF patients as failed uptake of NE by the myocardium can lead to decompensation and advanced CHF.

EAT is significantly associated with LV diastolic dysfunction in T2DM patients, which has been theorized to occur from reduced coronary flow due to compression or paracrine influence of vasoconstriction [126]. A further cross-sectional study found reduced diastolic and systolic function among diabetics with increased EAT [127]. While diastolic function was most commonly observed, studies that have assessed EF have described subclinical systolic derangement as well [113]. Increased EAT was correlated with LV diastolic relaxation velocity and left atrial enlargement on echocardiogram independent of BMI, which is in keeping with the atrial fibrotic remodeling and dilatation previously discussed in the context of AF [128]. T2DM patients with lower BMIs had a reduced level of EAT and improved cardiac functioning when assessed through cardiac CT, MRI, and other imaging [129]. Hence, there appear to be clear links between EAT and cardiac mechanistic functioning associated with T2DM and obesity.

Most studies have investigated the volume of EAT and its relation to diastolic dysfunction, but fewer have investigated the metabolic functioning of the tissue. It has been demonstrated that CHF is associated with a pathologic EAT phenotype due to disturbed energy homeostasis and adipokine production. Experimental models show that insulin resistance itself can lead to altered cardiac contractility [20,130]. Insulin resistance can also cause increased ROS due to greater fat oxidation [109] and an increase in myocardial triglyceride stores, which could be influenced by an elevated FFA output from EAT [131]. In keeping with the hypothesis of an altered EAT phenotype centering on the development of CVD, Pérez-Belmonte et al. demonstrated a reduced expression of thermogenic genes UCP1 and PGC1α in the EAT of patients with HFrEF as well [132]. Finally, mitochondrial dysfunction from T2DM contributes to CHF by altering the efficiency of ATP generation and calcium movement across the membrane [114]. Mitochondrial dysfunction in this case could be associated with the loss of thermogenesis and inefficient energy homeostasis [114].

**Figure 6:**
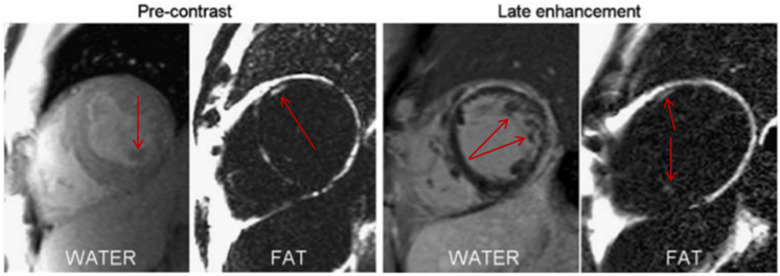
Fibrofatty infiltrates highlighted in fat-water separated MRI pre-contrast (**left**) and late enhancement (**right**) in a patient with non-ischemic cardiomyopathy. Reproduced from Kellman et al. [46] under the Creative Commons Attribution Noncommercial License.

Another interesting feature of LV diastolic dysfunction in T2DM patients is fibrofatty infiltrates. They form a key pathologic characteristic in many non-ischaemic cardiomyopathies, particularly in diabetic cardiomyopathy. Although echocardiography is an adequate imaging technique for EAT thickness [27], fat–water-separated MRIs are shown to have greater sensitivity in detecting intramyocardial fat and fibrofatty infiltrates [46]. An example of this is shown in Figure 6. This shows that the pathophysiologic roles undertaken by the EAT, such as myocardial infiltration, inflammation, and altered metabolism are key characteristics in the development of the CVDs investigated. While the clinical presentations of these conditions are widely varied, it is possible that these underlying mechanisms relate to the phenotype of EAT and its disturbed functioning.

## 4. Clinical Implications and Future Directions

From these studies, the association between EAT and CVD is evident, though some finer details are still emerging. The impact of lifestyle and pharmacologic therapies on the functioning of EAT is worth investigating in the context of re-browning. EAT has shown a reduction in response to antidiabetic drugs such as GLP-1A [133], SGLT-2 inhibitors [134], and metformin [135]. Statins have also been shown to reduce EAT volume [136,137,138]. In addition, treatment with statins reduced EAT and inflammation, as shown by CT. This effect of statins on EAT was independent of the LDL cholesterol-lowering effect of statins and did not affect subcutaneous fat [139]. While these studies show a reduction in volumes, a change in their phenotype from pro-inflammatory/pro-fibrotic or to more brown-like characteristics was not documented. Exercise is an important lifestyle factor. During exercise, myokines, such as fibroblast growth factor 21 (FGF21), are released during muscle contractions. FGF21 is an example of a potent browning agent that has shown promise in rodent models [18,140]. This, along with other myokines, could upregulate brown-like characteristics from EAT. Finally, diet-induced thermogenesis has been postulated in some literature, where dietary nitrates in green vegetables are thought to improve endothelial function and increase cyclic GMP involved in thermogenesis [141,142]. These are potential therapeutic routes that could be explored, but further research on EAT is warranted.

Most studies included in this review analyzed a relatively small sample size and many do not correct for confounding factors such as BMI, age, co-morbidities, and medications. Similarly, while cardiac MRIs are considered the gold standard in quantifying EAT, most studies opted for echocardiography, which allows mainly for a two-dimensional representation of the tissue. Studies have also noted the similarity of EAT to other visceral AT depots, making it difficult to attribute systemic metabolic effects to any one tissue. The studies show clear links between EAT and CVD, and that including its characteristics improved risk stratification of CVD patients. Each CVD investigated shows a multifaceted pathophysiologic mechanism involving EAT but linking back to the key characteristic: an altered functioning of the tissue. There is evidence of increased pro-inflammatory/pro-fibrotic signaling, ROS, and an overall shift in structural and secretory phenotype. However, further research on the exact cause of the shift in phenotype and a clearer picture of its downstream effects will be necessary before it can be used as a clinical tool.

## Figures and Tables

**Table 1:** Adipokines secreted by EAT and their protective/maladaptive roles.

Anti-Inflammatory, Protective Adipokines		Pro-Inflammatory, Pathologic Adipokines	
Factor	Effect	Factor	Effect
Adiponectin	Vasodilation, prevention of monocyte adhesion to endothelium, promotes local nitric oxide action [40].	TNF-α, IL-1, IL-6, IL-8	Released from immune cells and adipocytes. Induce lipolysis, promote apoptosis, inhibit adiponectin secretion, recruit nearby immune cells [25].
Leptin	Endothelium-dependent vasodilation [40].	Resistin	Released from macrophages and adipocytes. Released in response to TNF-α [25].
Omentin-1	Inhibits TGF-β and limits fibroblast differentiation and collagen deposition [25].	Visfatin	Acts as an insulin-mimetic and is a marker of visceral fat. Thought to play a role in vascular inflammation and remodeling [41].
Adrenomedullin	Antioxidant, angiogenic, vasodilatory, and anti-inflammatory properties. Inhibits nuclear factor kappa B (NF-κB) pathway involved in inflammation [25].	Leptin	Enhance smooth muscle cell proliferation, angiogenesis, platelet aggregation and increased leptin expression on atherosclerotic lesions suggest atherogenic role [42].
UCP1/PGC-1α	Proteins aid in mitochondrial release of heat through consumption of chemical energy–thermogenic capability [18].	Ang II	Promotes macrophage polarisation to inflammatory phenotype, promoting production of fibrotic cytokines (TGF-β), vasoconstriction [43].

## Abbreviations

AF = atrial fibrillation
AGE = advanced glycation end products
AP = action potential
CAC = coronary artery calcium
CAD = coronary artery disease
CD = cluster of differentiation
CHF = chronic heart failure
CT = computed tomography
CVD = cardiovascular disease
Cx = connexins
EAD/DAD = early/delayed after depolarizations
EAT = epicardial adipose tissue
ECM = extracellular matrix
EF = ejection fraction
EFV = epicardial fat volume
FFA = free fatty acids
HDL = high-density lipoprotein
IL = interleukin
LAA/RAA = left/right atrial appendages
LDL = low-density lipoprotein
LV/LA = left ventricle/atrium
MMP = matrix metalloproteinases
MS = metabolic syndrome
NEFA = non-esterified fatty acids
NLRP3 = leucine-rich repeat-containing receptor family pyrin domain-containing 3
PVAT = perivascular adipose tissue
SD = standard deviation
T2DM = type 2 diabetes mellitus
TGF = transforming growth factor
TNF = tumor necrosis factor
